# Cognition as mediator of pulmonary function and risk of sarcopenia among older adults

**DOI:** 10.1186/s12889-024-18848-5

**Published:** 2024-05-18

**Authors:** Zhao Hu, Lu Tang, Yiqiang Zhan

**Affiliations:** 1 Department of Epidemiology, School of Public Health (Shenzhen), Sun Yat-Sen University, Shenzhen, China; 2 The Seventh Affiliated Hospital, Sun Yat-Sen University, Shenzhen, China; 3 Institute of Environmental Medicine, Karolinska Institutet, Stockholm, Sweden

**Keywords:** Sarcopenia, Cognition, Lung function, Mediation, Cohort

## Abstract

**Background:**

The relationship between lung function and sarcopenia remains ambiguous. The primary aim of this study was to investigate the potential association between lung function and sarcopenia in the older adults, as well as to examine the mediating role of cognitive function in this relationship.

**Methods:**

The participants were selected from a nationally representative population-based cohort in China. The peak expiratory flow (PEF) measurement was used to evaluate the lung function in older persons. The sarcopenia was diagnosed using the guidelines of the Asian Working Group for Sarcopenia (AWGS) in 2019. The Cox proportional hazard model was utilized to perform primary analyses of the relationship between PEF and sarcopenia. The mediating effect of cognitive function was evaluated using the counterfactual mediation method.

**Results:**

This cohort study included 4,011 older adults (average age, 66.6 years; 53.3% males). During a follow-up period of 3.86 years, 349 individuals were diagnosed with sarcopenia. After adjusting for potential confounders, each one-standard-deviation increase in PEF was associated with a 28% reduction in the risk of sarcopenia (hazard ratio [HR]: 0.72; 95% confidence interval [CI]: 0.63, 0.80). There was a significant mediation of cognition for the association between PEF and incident sarcopenia, and the proportion mediated was 12.2% (95% CI: 4.5%, 23.1%).

**Conclusions:**

Older adults with impaired lung function are more likely to develop sarcopenia. Nevertheless, cognition can explain only a small portion of this association. Thus, other potential pathways between lung function and sarcopenia must be elucidated.

**Supplementary Information:**

The online version contains supplementary material available at 10.1186/s12889-024-18848-5.

## Introduction

According to the European Working Group on Sarcopenia in Older People (EWGSOP), sarcopenia is characterized as a gradual and widespread skeletal muscle condition linked to numerous negative health consequences [1]. Sarcopenia is becoming more common as individuals age, and it is more prevalent among older adults [2]. However, it has been acknowledged that they can develop earlier in life [3]. Sarcopenia affects approximately 10%∼27% of older persons individuals globally [4] and is linked to a higher risk of falls, fractures, physical impairment, and mortality [5]. Moreover, prior research has linked different aspects of lifestyle, including lack of exercise, poor diet, tobacco use, and excessive sleep, to a higher likelihood of developing sarcopenia [5, 6]. Nevertheless, these associations primarily rely on observational studies not conducted within a specific cohort.

Several prior observational investigations have indicated a potential link between sarcopenia and lung function in older adults. A Brazilian study discovered that spirometric variables, such as forced vital capacity (FVC), exhaled tidal volume, and forced expiratory volume in one second (FEV1), correlate negatively with sarcopenia [7]. Similarly, an Italian study found that FVC, FEV1, and peak expiratory flow (PEF) are positively associated with handgrip strength and chair stand tests [8]. The potential predictive role of pulmonary function on sarcopenia development, however, has not been explored in cohort studies.

Given the association between lung function and cognitive decline [9, 10] and the observed link between cognitive impairment and sarcopenia [6], cognitive function may mediate the association between lung function and sarcopenia. Previous research has indicated that the buildup of amyloid-β (Aβ) in neuronal tissue in old mice may play a role in muscle atrophy and disruption of neuromuscular junctions(NMJ), which as the nexus between the nervous and muscular systems, is critical for input and dependable neural control of muscle force generation [11]. Additionally, pulmonary impairment has been linked to deficits in social recognition memory and changes in microglial profiles within the suprachiasmatic nucleus of the hypothalamus [12]. In instances of prolonged decline in pulmonary function, individuals may experience hypoxic conditions, which can have detrimental effects on neuronal oxygen homeostasis [13], may contributed to the onset and progression of cognitive impairment [14]. Furthermore, systemic inflammation and immune dysregulation associated with certain pulmonary diseases may lead to neuroinflammation in the central nervous system, facilitated by the infiltration of proinflammatory cytokines across the blood-brain barrier [15]. These modifications in neurological factors may be linked to alterations in NMJ, such as changes in the structure and arrangement of pre- and postsynaptic membranes, a decrease in the quantity of synaptic vesicles containing neurotransmitters, and a deceleration in axonal transport, which may play a role in the development of sarcopenia [16, 17]. Currently, no observational study has evaluated cognition as a mediator or modifier of the association between pulmonary function and the risk of sarcopenia.

This study aimed to investigate the association between a measure of respiratory capacity, specifically PEF, and the likelihood of sarcopenia in older adults using a large prospective cohort study. Moreover, we evaluated whether cognition mediates the association between pulmonary function and sarcopenia.

## Method

### Study population

In this study, participants were selected from the nationally representative study sample, the China Health and Retirement Longitudinal Study (CHARLS). CHARLS aims to collect precise and dependable micro-data concerning households and individuals aged 45 years or older in China. These data were used to analyze and address the population-aging problem in the country. In 2011, the national baseline survey called CHARLS was launched, involving 150 units at the county level, 450 units at the village level, and approximately 17,000 individuals from 10,000 households. The samples were monitored at two- to three-year intervals to observe any alterations. Individuals were interviewed at their residences using computer-assisted personal interviewing technology. More detailed information about CHARLS has been described in the previous literature [18]. The CHARLS survey project was approved by the Biomedical Ethics Committee of Peking University, and informed consent was signed by participants.

This study used data from three waves collected in 2011 (baseline), 2013, and 2015. The sample size was 17,708 at baseline. Participants were excluded due to age less than 60 years (*n* = 9,982) or without age information (*n* = 60), without physical measurement data for determining sarcopenia at baseline (*n* = 2,521), diagnosed with sarcopenia according to AWGS2019 criteria at baseline (*n* = 808), no information on lung function (*n* = 88), no information on cognition (*n* = 184), and missing covariate data (*n* = 54). Finally, 4,011 eligible individuals were included in this prospective study. e-Figure S1 illustrates a detailed flowchart of the sample selection.

### Measurement of lung function

The PEF measurement at baseline(2011) was used to evaluate the lung function of older persons. PEF is a dependable measure of lung function and is defined as the highest flow achieved during maximal expiration from maximum lung inflation [19]. It strongly associates with spirometry-measured FEV1 and provides a more accurate indication of airway patency [20]. In the CHARLS study, PEF was measured in L/min by well-trained technicians using a peak flow meter equipped with a disposable mouthpiece (EverpureTM; Shanghai, China). The examiner thoroughly inspected and calibrated the equipment before the examination, comprehensively explaining the measurement procedures. Participants were instructed to rise, inhale deeply, position their lips on the mouthpiece, and exhale forcefully and rapidly. Scientists documented the needle’s reading and recalibrated the meter for two additional measurements. In this study, a timer was employed to establish a 30-s duration between each measurement, and the maximum values from the three tests were analyzed. The predicted normal value of PEF was calculated using a formula from a previous study in Chinese adults [21], considering age, gender, and height. Peak expiratory flow rate (PEFR) was calculated by dividing the actual value by the predicted value.

### Measurement of cognition function

Trained investigators evaluated cognitive function at baseline(2011) through in-person interviews covering four aspects: orientation, episodic memory, computation, and drawing [22]. The orientation aspect included five elements: year, month, day, weekday, and the present season. A single point was given to each item for correct participant responses, resulting in a cumulative score of five points. Participants were assessed on their computational skills by subtracting seven from 100 five times in a row, with one point for each accurate answer. Episodic memory consists of both immediate and delayed word recalls. Participants were given a set of 10 words and instructed to remember as many words as possible promptly.The scores for immediate recall ranged from 0 to 10, with a score of 1 for every word that was remembered accurately. Delayed recall was assessed using a comparable scoring criterion. Drawing ability was assessed by testing the ability to redraw a previously displayed picture and was scored from 0 to 1. Cognitive function was evaluated by adding scores for orientation(5 points), computation(5 points), episodic memory(20 points), and drawin(1 point), resulting in a maximum score of 31. Higher scores indicate better cognitive function.

### Outcome: sarcopenia assessment

Sarcopenia was evaluated at each wave(2011,2013 and 2015) using the Asian Working Group for Sarcopenia in 2019 (AWGS2019) standards [2], including muscle strength, appendicular skeletal muscle mass (ASM), and physical performance. According to the AWGS2019 recommendations, muscle strength is expressed as handgrip strength. In CHARLS, handgrip strength was measured twice for each hand using the YuejianTM WL-1000 dynamometer held at a 90° angle. The mean of the accessible data on the maximum strength was utilized. According to the AWGS 2019 diagnostic criteria, females with low handgrip strength are defined as having less than 18 kg and males with less than 28 kg [2]. Based on a previously validated formula, ASM estimation was conducted in the Chinese population [23]. This formula demonstrated strong concordance with dual-energy X-ray absorptiometry (DXA) [23, 24]. Among the study population, low muscle mass was defined as the bottom 20% of sex-specific height-adjusted muscle mass (ASM/height^2^) [25]. In this research, the initial measurement indicated that the threshold for low muscle mass was below 6.80 kg/m^2^ for males and below 4.97 kg/m^2^ for females. According to AWGS 2019, physical performance was assessed by measuring gait speed, conducting the five-time chair stand test, and administering the short physical performance battery (SPPB). The participants in the gait speed test were directed to walk 2.5 m twice (round trip) at their usual pace. The completion time for this task was recorded. The five-time chair stand test assessed the time it took for a participant to stand up from a chair height of 47 cm, with arms crossed over the chest, five consecutive times. The SPPB assessment consisted of three position tests, with each test lasting for 10 s: (1) side-by-side position, (2) semi-tandem position, and (3) tandem position. Each SPPB test contributed 4 points, resulting in a total score of 12 points. In accordance with AWGS 2019 criteria, low physical performance was defined as gait speed of < 1.0 m/s, five chair standing tests ≥ 12 s, or SPPB score ≤ 9 [2]. The same instruments and procedures were used for all three waves. The instruments and details of each measurement are available in the CHARLS study overview [18]. Based on the AWGS2019 diagnosis criteria, sarcopenia was defined as low ASM and either low muscle strength or low physical performance.

### Covariates

According to prior knowledge, potential covariates included demographic information (age, gender, education, and marital status), lifestyle behaviors (smoking, alcohol consumption, sleep duration, and nap duration), and health conditions (hypertension, type 2 diabetes, dyslipidemia, chronic lung diseases, cardiovascular disease (CVD), and arthritis or rheumatism). The detailed assessment methods and definitions are provided in Supplemental material eMethod1.

### Statistical analyses

The mean and standard deviation (SD) were used to present descriptive characteristics at baseline for quantitative variables, while frequencies and percentages were used to present categorical variables. The differences between the three PEF tertile categories were tested using Analysis of Variance (ANOVA) or the chi-square test.

Cox proportional hazards models was fitted for examine the association between PEF, PEFR, and sarcopenia. The proportional hazard assumption was tested using the Schoenfeld residuals. The results were reported as hazard ratios (HRs) and 95% confidence intervals (CIs). Follow-up time was used as a time-dependent variable. Nonlinear associations between lung function indicators and sarcopenia were determined using restricted cubic splines (RCS) fitting Cox proportional hazard models. Knots between 3 and 7 were tested, and the model with the lowest Akaike information criterion value was selected for the RCS. The mean value of each exposure was used as the reference group for the curves.

The association between lung function indicators and sarcopenia and the mediating effect of cognitive function were tested using the counterfactual mediation method [26]. Two separate models were fitted to estimate the effect size of the mediation. The Cox proportional hazard model was first used to predict sarcopenia based on exposure (lung function indicators), mediator (cognition function), an interaction term between exposure and mediator, and confounders. The mediator (cognition function) was second modeled using linear regression models based on exposure and confounders. All models were adjusted for age, gender, education, marital status, smoking, alcohol consumption, sleep duration, nap duration, hypertension, diabetes, dyslipidemia, chronic lung diseases, CVD, and arthritis. The directed acyclic graph is illustrated in eFigure 2. The total effect of lung function indicators on incident sarcopenia decomposes into four ways (eFigure 3). The total association of lung function indicators with incident sarcopenia was decomposed into controlled direct association (CDA), reference interaction (INTref), pure indirect association (PIA), and mediated interaction (INTmed) [27]. The proportion of the association between lung function indicators and sarcopenia mediated through cognitive function was calculated as the sum of the percentage of excess association of PIA and INTmed [28].

Several sensitivity analyses were performed: (1) adding a lag time (a two-year lag time in this study) between lung function and cognition function (the exposure and confounders of baseline while the mediator of the second minimum); (2) excluding participants who self-reported suffering from chronic lung disease at baseline (*n* = 515); (3) excluding participants who had PEF values less than 60 L/min at baseline (*n* = 104); (4) excluding all participants who experienced events within the first two years of follow-up to minimize the effect of reverse causality (*n* = 182). A subgroup analysis stratified by gender was also performed. All analyses were conducted using Stata 17.0 (College Station, Texas 77,845 USA) and R statistical software version 4.2.3 (R Project for Statistical Computing).

## Results

Table 1 presents the characteristics of the population according to tertiles of PEF. The participant’s average age was 66.6 years, and 53.3% were male. The average follow-up duration was 3.86 years (SD: 0.51 years). Over this period, 349 participants were diagnosed with sarcopenia using the AWGS2019 criteria. Overall, participants with a higher PEF were younger, more likely to be male, had higher cognition scores, without hypertension, chronic lung diseases, and CVD.

**Table 1:** Characteristics of the population by tertiles of peak expiratory flow

Characteristics	Total (*n* = 4011)	Lowest (*n* = 1355)	Middle (*n* = 1293)	Highest (*n* = 1363)	*P*-value ^a^
Age (years), mean (SD)	66.6 (5.6)	67.8 (6.1)	66.4 (5.6)	65.5 (5.0)	< 0.001
Gender (male), *n* (%)	2137 (53.3)	530 (39.1)	564 (43.6)	1043 (76.5)	< 0.001
Education, *n* (%)					< 0.001
Primary school and below	3184 (79.4)	1172 (86.5)	1077 (83.3)	935 (68.6)	
Middle school	559 (13.9)	132 (9.7)	158 (12.2)	269 (19.7)	
High school	268 (6.7)	51 (3.8)	58 (4.5)	159 (11.7)	
Marital status (married), *n* (%)	3334 (83.1)	1074 (79.3)	1064 (82.3)	1196 (87.7)	< 0.001
Smoking, *n* (%)	1268 (31.6)	323 (23.8)	379 (29.3)	566 (41.5)	< 0.001
Alcohol drinking, *n* (%)	1019 (25.4)	287 (21.2)	269 (20.8)	463 (34.0)	< 0.001
Sleep duration (hours), *n* (%)					0.043
< 7	2142 (53.4)	742 (54.8)	702 (54.3)	698 (51.2)	
7–8	1530 (38.1)	489 (36.0)	478 (37.0)	563 (41.3)	
> 8	339 (8.5)	124 (9.2)	113 (8.7)	102 (7.5)	
Nap duration (minute), *n* (%)					0.021
0 ∼ 30	2096 (52.3)	736 (54.3)	679 (52.5)	681 (50.0)	
31 ∼ 60	1140 (28.4)	393 (29.0)	360 (27.8)	387 (28.4)	
61∼	775 (19.3)	226 (16.7)	254 (19.6)	295 (21.6)	
Cognition score, mean (SD)	13.2 (5.6)	11.5 (5.5)	12.9 (5.6)	15.0 (5.2)	< 0.001
Hypertension, *n* (%)	2216 (55.2)	814 (60.1)	716 (55.4)	686 (50.3)	< 0.001
Type 2 diabetes, *n* (%)	1593 (39.7)	518 (38.2)	528 (40.7)	549 (40.3)	0.380
Dyslipidemia, *n* (%)	2365 (59.0)	781 (57.6)	756 (58.5)	828 (60.7)	0.234
Chronic lung diseases, *n* (%)	515 (12.8)	282 (20.8)	148 (11.4)	85 (6.2)	< 0.001
Cardiovascular disease, *n* (%)	697 (17.4)	297 (21.9)	216 (16.7)	184 (13.5)	< 0.001
Arthritis or rheumatism, *n* (%)	1481 (36.9)	510 (37.6)	494 (38.2)	477 (35.0)	0.184
PEF (L/min), mean (SD)	269.8 (119.7)	143.9 (48.1)	260.4 (31.3)	403.8 (71.4)	< 0.001
PEFR, mean (SD)	0.8 (0.3)	0.4 (0.2)	0.8 (0.2)	1.1 (0.2)	< 0.001
Sarcopenia during follow-up, n(%)	349 (8.7)	162 (12.0)	117 (9.0)	70 (5.1)	< 0.001

^a^*P*-value was examined using ANOVA or Chi-square test

SD, standard deviation; PEF, peak expiratory flow; PEFR, peak expiratory flow rate

**Fig. 1:**
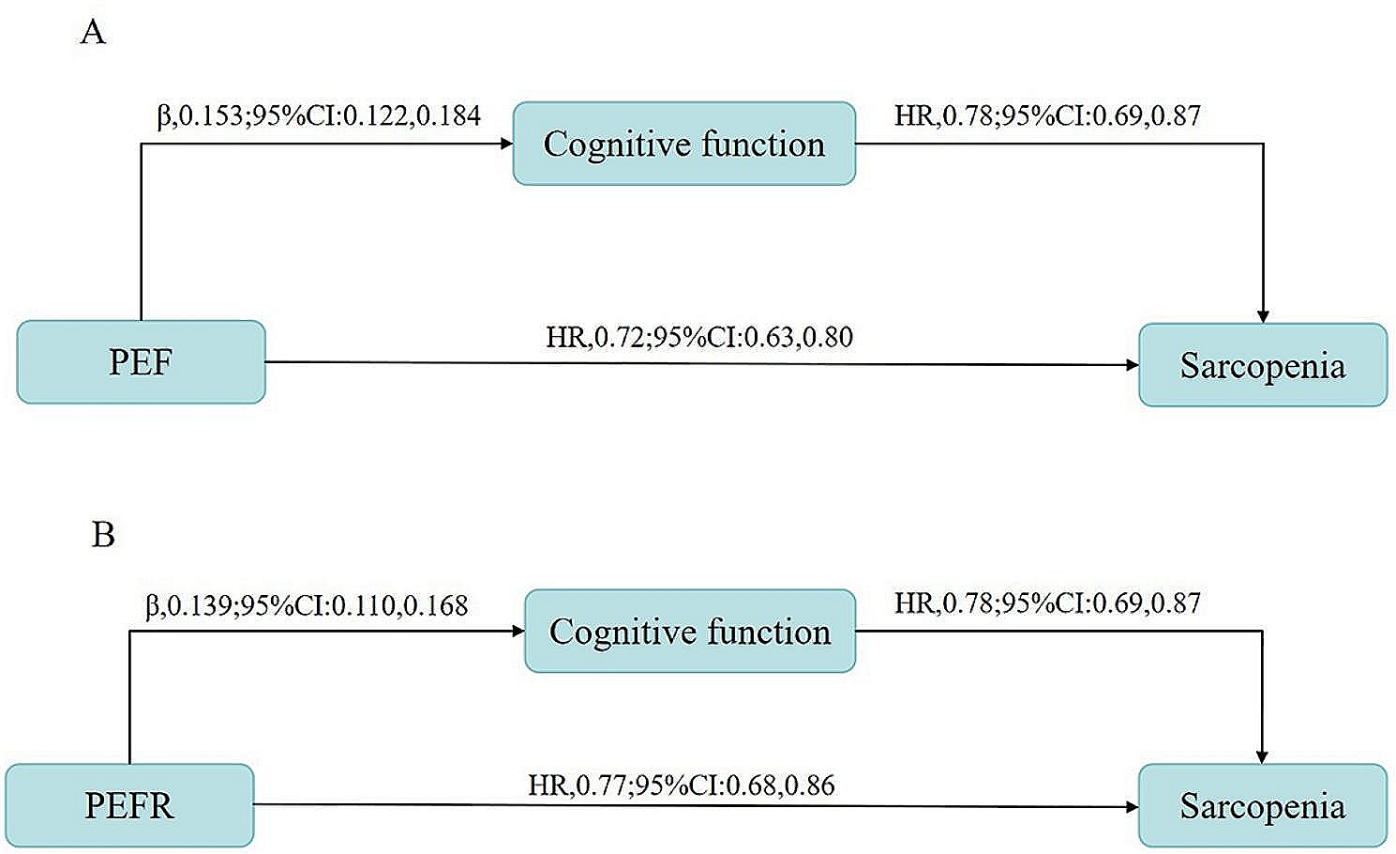
Cognitive function acts as a mediator in the relationship between lung function indicators and sarcopenia. **Panel A**: Cognitive function as a mediator for the association between PEF and sarcopenia. **Panel B**: Cognitive function as a mediator for the association between PEFR and sarcopenia Note: The association of per SD increment in PEF or PEFR with sarcopenia was fitted using Cox proportional hazard models after adjusting for age, gender, education, marital status, smoking, alcohol consumption, sleep duration, nap duration, hypertension, diabetes, dyslipidemia, chronic lung diseases, cardiovascular disease, and arthritis. The association between PEF or PEFR and cognition function was modeled using generalized linear regression after adjusting for the same covariates, whereas the association of per one SD increment in cognition function (5.6 points) with sarcopenia was modeled using Cox proportional hazard regression was also adjusted for PEF or PEFR PEF, peak expiratory flow; PEFR, peak expiratory flow rate

Figure 1 exhibits an association between the lung function markers and sarcopenia risk. After adjusting for all covariates, we found that each SD increment in PEF was associated with a lower 28% risk of sarcopenia among older adults (HR, 0.72; 95% CI: 0.63, 0.80). Finally, we fitted a three-knots RCS for a non-linear association between lung function indicators and sarcopenia risk. eTable 1 lists the Akaike information criteria for each knot of the RCS. eFigure 4 and eFigure 5 present non-linear associations between lung function indicators and the risk of sarcopenia. After adjusting for covariates, three-knots RCS exposed that higher PEF was associated with a lower risk of sarcopenia (*P* for overall < 0.001), without evidence of non-linearity between PEF and risk of sarcopenia (*P* for non-linear = 0.079).

Table 2 shows the counterfactual mediation methods results for the association between lung function indicators and sarcopenia, with cognitive function as the mediator. The adjusted HR for the total association of PEF with risk of sarcopenia was 0.70 (95% CI: 0.62, 0.79). There is no evidence of an interaction between cognition function and PEF via the INTref or INTmed path, and most of the associations were direct. There was significant mediation of cognition function for the association between PEF and incident sarcopenia, with a proportion of 12.2% (95% CI: 4.5%, 23.1%), whereas it was 16.2% (95% CI: 3.0%, 49.1%) in females and 11.4% (95% CI: 2.1%, 29.9%) in males (eTables 2 and 3). When PEFR was used as the exposure, similar results were obtained.

**Table 2:** Mediation role of cognitive function for the association between lung function indicators and sarcopenia

Association component	PEF		PEFR	
	HR (95% CI)^a^	Percentage of excess association (95% CI)	HR (95% CI)	Percentage of excess association (95% CI)
Total association	0.70 (0.62, 0.79)	100	0.76 (0.68, 0.84)	100
Controlled direct association	0.72 (0.63, 0.80)	88.3 (79.5, 96.1)	0.77 (0.68, 0.86)	88.4 (74.5, 99.8)
Reference interaction	0.00 (-0.03, 0.04)	–0.5 (–13.3, 8.9)	0.01 (–0.02, 0.05)	–3.6 (–24.1, 8.0)
Mediated interaction	0.00 (-0.01, 0.02)	–1.2 (–5.5, 3.0)	0.00 (–0.01, 0.01)	0.1 (–4.3, 6.5)
Pure indirect association	0.96 (0.94, 0.98)	13.4 (7.0, 21.3)	0.96 (0.94, 0.98)	15.1 (7.8, 28.0)
Proportion mediated (%)	12.2 (4.5, 23.1)		15.2 (7.1, 31.4)	

^a^ adjusted for age, gender, education, marital status, smoking, alcohol consumption, sleep duration, nap duration, hypertension, diabetes, dyslipidemia, chronic lung diseases, cardiovascular disease, and arthritis

PEF, peak expiratory flow; PEFR, peak expiratory flow rate; HR, hazard ratio; CI, confidence interval

Sensitivity analysis results displayed that the proportion mediated was 10.3% (95% CI: 4.3%, 20.1%) when a two-year lag time of cognitive function was a mediator for the total association between PEF and incident sarcopenia (eTable 4). Similar results were obtained after excluding participants who self-reported chronic lung disease (eTable 5) or PEF < 60 L/min at baseline (eTable 6). The mediation of cognitive function was non-significant after excluding sarcopenia events within the first two years of follow-up (eTable 7).

## Discussion

This study found that higher PEF was associated with better cognition function in older adults at baseline. Furthermore, better cognition was associated with a lower risk of sarcopenia. Finally, we observed that a higher PEF was associated with a lower risk of incident sarcopenia, and cognition explained that the association between PEF and sarcopenia was small.

Our findings regarding lung and cognitive function at baseline are consistent with prior research [29, 30]. Previous studies have linked decreased lung function to poor cognitive function and dementia [31, 32]. Moreover, decreased pulmonary function is more likely to predict cognition decline [33]. Pulmonary function decline may decrease cognitive performance via hypoxia, decreased neurotransmitter function, increased systemic inflammatory processes, or a combination of various factors [9, 10]. Although numerous research have examined that sarcopenia may be a potential contributing factor to cognitive impairment [34, 35], our findings indicate a positive correlation between diminished cognitive abilities and increased susceptibility to sarcopenia. Likewise, a longitudinal study also found that any cognitive impairment predicted decreased handgrip strength [36]. These data align with the findings of several cognitive training studies that have demonstrated a beneficial impact on physical performance [37, 38]. However, the underlying mechanisms between cognition and skeletal muscle have not been fully elucidated, and the muscle-brain relationship warrants further investigation.

Consistent with prior observational research, our investigation discovered a positive correlation between enhanced respiratory function and a reduced likelihood of sarcopenia [39, 40]. Even in subgroup analysis among the self-reported chronic lung disease population, residual lung function was associated with sarcopenia. Earlier research has discovered that chronic obstructive pulmonary disease(COPD)patients with sarcopenia exhibit reduced maximal inspiratory pressure and diminished respiratory muscle strength [41, 42]. However, no study has evaluated the mediating effect of cognition on lung function in sarcopenia using formal mediation analysis.

Our study presented that approximately one-tenth of the association between poor lung function and incident sarcopenia contributed to poor cognition, with most of the association attributed to the direct effect of poor lung function. An alternative behavioral mechanism is that aging weakens the respiratory system, a person’s physical capacity decreases as they age because they become less tolerant of physical exertion and become physically inactive. This may affect the musculoskeletal system with reduced strength and even loss of muscle mass.Another explanation is that chronic hypoxia, such as reduced PEF, may be associated with increased elastic fiber proteolysis and collagen, resulting in pulmonary hyperinflation. Thus, the oxygen supply transported to the muscles may be insufficient for proper functioning, resulting in sarcopenia development [43]. This can explain the high prevalence of sarcopenia in individuals with COPD [44]. Therefore, lung function may be an independent predictive factor for sarcopenia. A recently coined term called ‘Respiratory Sarcopenia’ or ‘Presbypnea’ refers to a condition where the body experiences sarcopenia, reduced respiratory muscle mass, decreased respiratory muscle strength, and/or impaired respiratory function [45]. Further studies are required to investigate the potential increase in the sensitivity and specificity of sarcopenia screening by incorporating additional indicators of lung function.

While a partial mediation effect was observed in the association between lung function and sarcopenia in this study, there are numerous factors that may have influenced this finding, indicating the need for further research. It should be noted that cognitive function was self-reported in this study, potentially introducing measurement bias, and a one-point difference in cognitive function score within this association may be considered minor. Additionally, the co-occurrence of sarcopenia and cognitive decline in older individuals has been well-documented in the literature [46]. Declining muscle function accelerates cognitive impairment, and cognitive impairment in turn affects muscle strength [47]. Additionally, various neurological factors related to cognitive function may act as mediators in this relationship. The impairment of lung function, linked to neuroendocrine connections involving testosterone, insulin, and growth factors, exerts significant impacts on both muscle and brain function [48]. A recent study has identified a potential association between sarcopenia and cognitive function in older individuals, suggesting a potential mechanism involving muscle-derived signaling molecules known as myokines in facilitating communication between muscle and brain tissues [49].

Extensive studies have produced convincing proof regarding the effectiveness of exercise treatments in individuals with sarcopenia, although the results were inconsistent. Several studies have indicated that resistance training can enhance muscle strength, skeletal muscle mass, and physical function [50, 51], whereas aerobic exercise augments the cross-sectional areas of muscle fibers [52, 53]. However, age-related decline in lung function cannot be reversed through training. Decreased pulmonary function likely contributes to exercise intolerance in healthy older adults, particularly those who maintain physical activity during senescence [54]. A multi-component or appropriate exercise therapy plan may be optimal. From our results and the viewpoint of prevention, fitted aerobic or balance exercises, such as walking in older adults, to retain aerobic capacity are useful, considering the ability to increase cerebral oxygenation and improve cognition function [55, 56].

Although we postulated that cognitive function could act as a mediator in altering the link between lung function and sarcopenia, other alternative pathways may influence the relationship between lung function and sarcopenia. Pulmonary limitation could potentially increase the likelihood of systemic inflammation [57]. However, chronic inflammation governs the development of age-associated sarcopenia [58]. In patients with COPD, handgrip strength and skeletal muscle mass index were correlated with IL-6 and TNF [59]. Moreover, there is a suggestion that unhealthy lifestyle behaviors, such as smoking and a poor diet, which are known to be linked to decreased lung function [60], may act as mediators in the relationship between lung function and sarcopenia, given their associations with sarcopenia [61]. Other pathways, such as depression, associated with both lung function and sarcopenia [19, 62], may play a role as a mediator and should be explored in further studies.

To our knowledge, this is the first study to evaluate the mediation role of cognition in the association between lung function and sarcopenia incidence in an older adult cohort. In contrast to prior cross-sectional research, our study examined the impact of lung function on the risk of sarcopenia and investigated its potential mechanism, providing evidence for preventing sarcopenia in older adults. However, our study has several limitations to address. First, unlike the ASM measurement method in AWGS or EWGSOP, namely DXA or bioelectrical impedance analysis (BIA), our study used a formula to estimate ASM, which is relatively insensitive to ASM longitudinal changes, leading to misclassification bias. However, the estimated value has good agreement with DXA. Second, the feasibility of spirometry is challenged in subjects with severe cognitive impairment [63], indicating that some participants may be excluded from PEF measurement, thereby increasing the possibility of miscalculating the association. Moreover, we did not evaluate the association of other lung function indicators, such as FEV, with incident sarcopenia. Third, there is a possibility of residual confounding due to the absence of other potential confounding factors, such as physical activity, which is why this study was unable to exclude residual confounding. Finally, we did not have lung function as a time-varying exposure since lung function may decline with aging and happen simultaneously with cognition decline because the limited mediation methods currently.

## Conclusion

Older adults with impaired lung function are more likely to develop sarcopenia. However, cognition function can explain only a small portion of this association. Therefore, additional studies on the pathways underlying the association between lung function and sarcopenia in older adults are needed. Moreover, targeted intervention strategies are necessary to slow lung function and prevent sarcopenia.

### Electronic supplementary material

Below is the link to the electronic supplementary material.

Supplementary Material 1

## Data Availability

Sequence data that support the findings of this study have been deposited in the http://charls.pku.edu.cn/.
