# Supplementary material for: Cognition as mediator of pulmonary function and risk of sarcopenia among older adults
Source: BMC Public Health. 2024 May 18;24:1347. doi: 10.1186/s12889-024-18848-5 (PMC11102626; doi:10.1186/s12889-024-18848-5)
Supplement: Supplementary file 1 — Supplementary Material 1 [file 12889_2024_18848_MOESM1_ESM.docx]

**Supplementary material**

**eMethod1 Covariates measurement**

Self-reported education was evaluated using a single inquiry asking about the highest level of educational attainment. The participants were categorized into primary school and below, middle school, and high school. Marital status was self-reported using the following question: What is your marital status currently? Participants were classified as married or unmarried. The married status encompasses being married with a spouse present but temporarily not living with a spouse due to reasons like work. A nonmarried status encompasses individuals who are separated, divorced, widowed or have never been married. Smoking was defined as the consumption of at least one cigarette per day. Drinking was defined as drinking an alcoholic beverage more than once per month. The participants were asked a single question to assess their sleep duration. The question was: 'During the past month, how many hours of actual sleep did you get at night (average hours for one night)?' Then, the sleep duration was categorized into three groups: less than 7 h, 7–8 h, and more than 8 h. The nap duration was evaluated by posing a question: During the past month, how long did you nap after lunch? The participants were divided into three groups: 0–30 min, 31–60 min, and 61 min or more. Hypertension was defined as hypertension diagnosed by a doctor or having a systolic blood pressure of 140 mmHg or higher, diastolic blood pressure of 90 mmHg or higher or being on medication to lower blood pressure. Type 2 diabetes was characterized by either self-reported doctor-diagnosed type 2 diabetes, fasting blood glucose levels of 7 mmol/L or higher, glycosylated hemoglobin (HbA1c) levels of 6.5% or above, or the use of antidiabetic medication. Dyslipidemia was characterized by triglycerides (TG) equal to or greater than 150 mg/dL, total cholesterol (TC) equal to or greater than 240 mg/dL, high-density lipoprotein cholesterol (HDL-C) equal to or greater than 40 mg/dL, and low-density lipoprotein cholesterol (LDL-C) equal to or greater than 160 mg/dL. Self-reported chronic lung diseases, including chronic bronchitis and emphysema, were diagnosed by doctors, excluding tumors or cancers. Cardiovascular diseases were assessed by self-report, including stroke, heart attack, coronary heart disease, angina, congestive heart failure, and other heart problems. Arthritis or rheumatism were assessed by self-report.

**eFigure 1 The flowchart of included participants in this study**


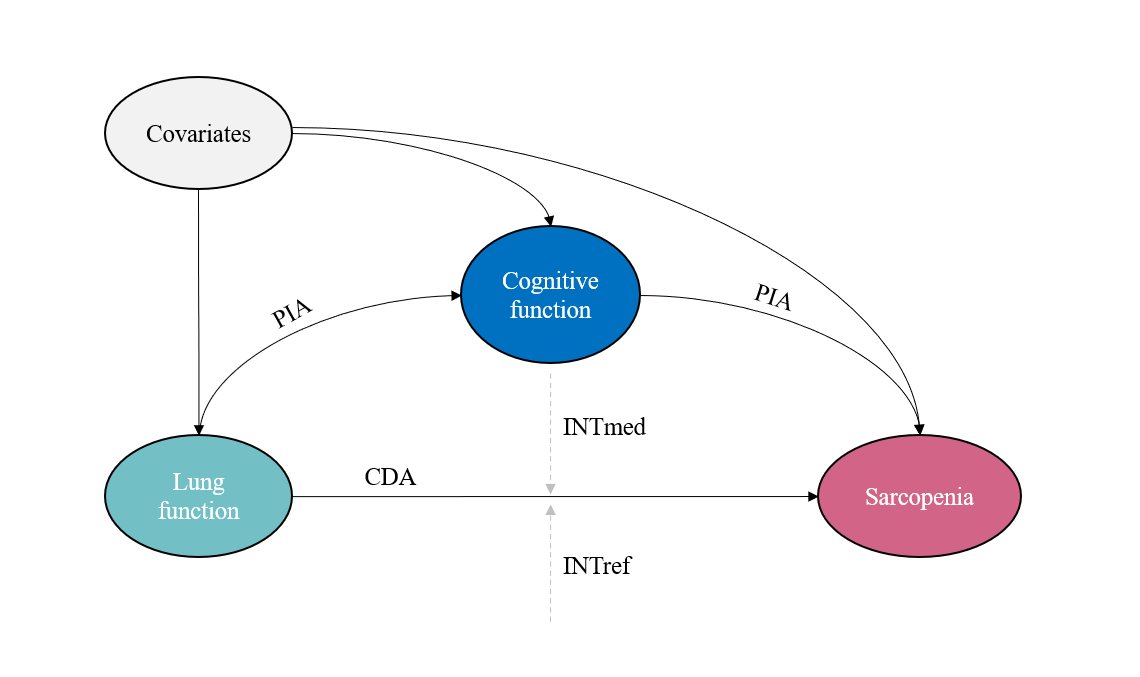


**eFigure 2. Directed acyclic graph between lung function indicators and risk of sarcopenia medicated by cognition function.**

Note: The total association of lung function indicators with incident sarcopenia was divided into controlled direct association (CDA), reference interaction (INTref), pure indirect association (PIA), and mediated interaction (INTmed). CDA is due to neither mediation nor interaction and represents the direct association between lung function and sarcopenia risk. The INTref is only due to interaction, representing the association between lung function and incident sarcopenia, and is modified by cognition function, but cognition function is not associated with lung function. The PIA is only due to mediation, representing the association of cognition function with incident sarcopenia, where cognition function is associated with lung function. INTmed is due to both mediation and interaction, indicating that the association between lung function and incident sarcopenia is modified by cognition function, and cognition function is associated with lung function. The covariates were age, gender, education, marital status, smoking, alcohol consumption, sleep duration, nap duration, hypertension, diabetes, dyslipidemia, chronic lung disease, cardiovascular disease, and arthritis.


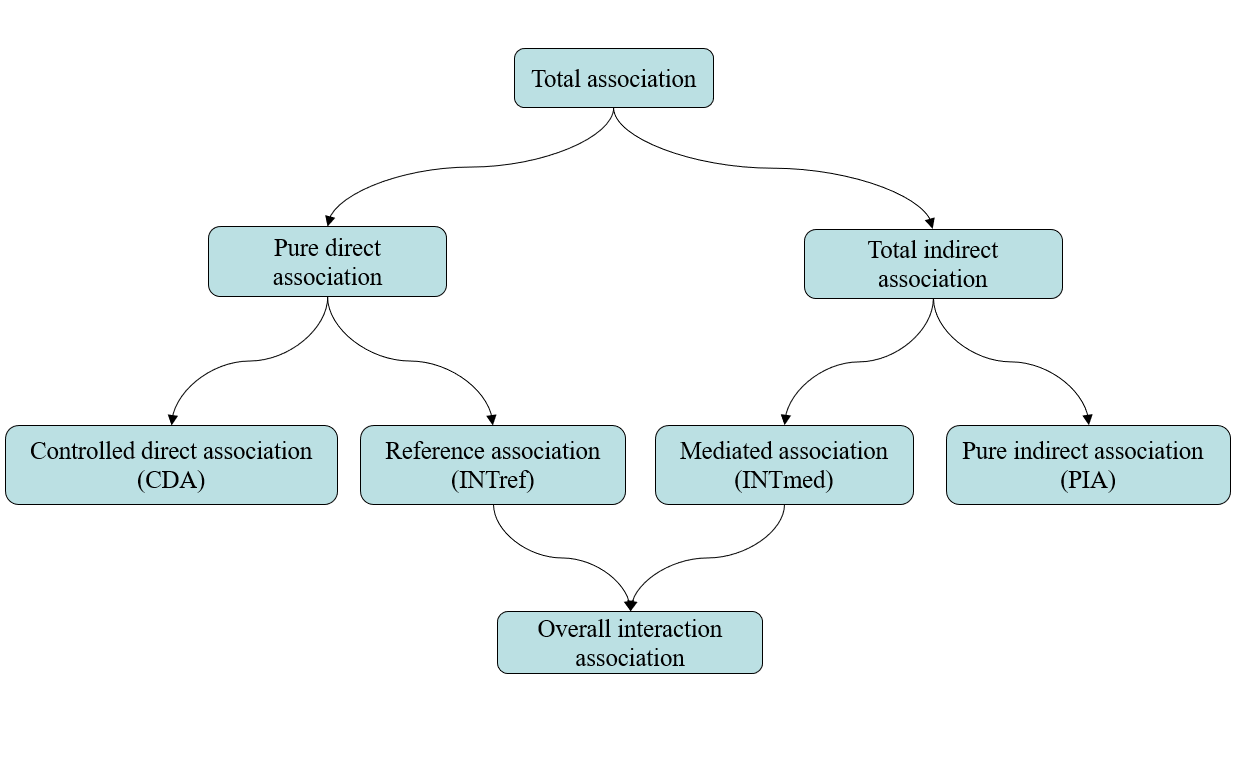


**eFigure 3. The 4-way decomposition of the total association.**

Note: CDA is due to neither mediation nor interaction; INTref is only due to interaction; PIA is only due to mediation; and INTmed is due to both mediation and interaction.

The pure direct association is the sum of CDA and INTref. The total indirect effect was the sum of INTmed and PIA, and the overall interaction effect was the sum of INTref and INTmed.


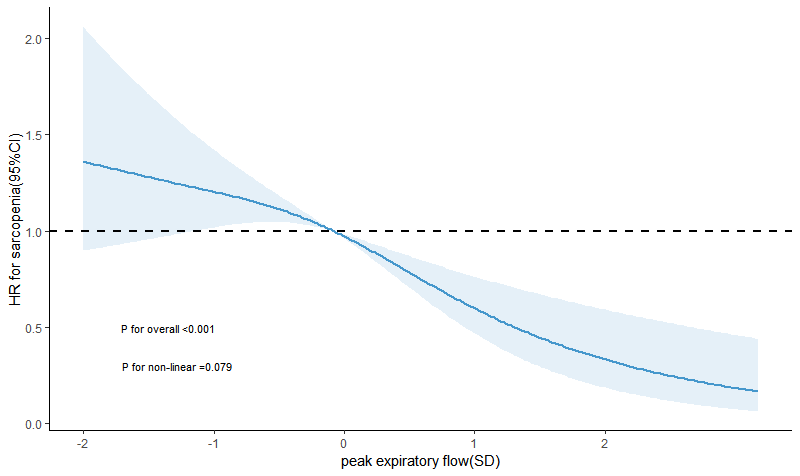


**eFigure 4 The non-linear association between peak expiratory flow and sarcopenia.**

**Note:** adjusted for age, gender, education, marital status, smoking, alcohol consumption, sleep duration, nap duration, hypertension, diabetes, dyslipidemia, chronic lung diseases, cardiovascular disease, and arthritis.

**
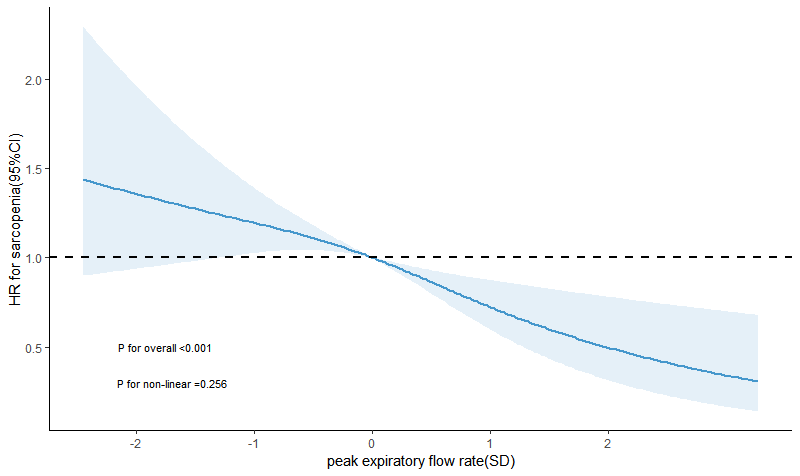
**

**eFigure 5 The non-linear association between peak expiratory flow rate and sarcopenia.**

**Note:** adjusted for age, gender, education, marital status, smoking, alcohol consumption, sleep duration, nap duration, hypertension, diabetes, dyslipidemia, chronic lung diseases, cardiovascular disease, and arthritis.

**eTable 1 Akaike information criterion for RCS when knots were selected from 3 to 7**

| Indicator | Knots=3 | Knots=4 | Knots=5 | Knots=6 | Knots=7 |
| --- | --- | --- | --- | --- | --- |
| PEF | 5581.604 | 5583.461 | 5585.470 | 5587.431 | 5589.377 |
| PEFR | 5589.178 | 5590.788 | 5592.666 | 5593.68 | 5596.258 |

PEF, peak expiratory flow; PEFR, peak expiratory flow rate

**eTable 2 Cognition function as mediator for the association between lung function indicators and sarcopenia in male older adults**

| Association component | PEF | |  | PEFR | |
| --- | --- | --- | --- | --- | --- |
|  | HR(95%CI)^a^ | Percentage of excess association (95% CI) |  | HR(95%CI)^a^ | Percentage of excess association (95% CI) |
| Total association | 0.75(0.65,0.85) | 100 |  | 0.79(0.68,0.90) | 100 |
| Controlled direct association | 0.76(0.65,0.87) | 92.4(80.5,100.8) |  | 0.80(0.70,0.92) | 90.9(71.6,103.7) |
| Reference interaction | 0.01(-0.01,0.05) | -3.8(-20.3,4.5) |  | 0.01(-0.01,0.05) | -5.6(-31.6,6.2) |
| Mediated interaction | 0.00(-0.02,0.01) | 0.9(-4.8,10.1) |  | 0.00(-0.02,0.01) | 1.6(-5.3,13.5) |
| Pure indirect association | 0.97(0.95,1.00) | 10.5(2.0,23.2) |  | 0.97(0.95,1.00) | 13.2(2.4,32.7) |
| Proportion mediated(%) | 11.4(2.1,29.9) | |  | 14.8(1.7,38.1) | |

^a^ adjusted for age, gender, education, marital status, smoking, alcohol consumption, sleep duration, nap duration, hypertension, diabetes, dyslipidemia, chronic lung diseases, cardiovascular disease, and arthritis.

PEF, peak expiratory flow; PEFR, peak expiratory flow rate; HR, hazard ratio; CI, confidence interval.

**eTable 3 Cognition function as mediator for the association between lung function indicators and sarcopenia in female older adults**

| Association component | PEF | |  | PEFR | |
| --- | --- | --- | --- | --- | --- |
|  | HR(95%CI)^a^ | Percentage of excess association (95% CI) |  | HR(95%CI)^a^ | Percentage of excess association (95% CI) |
| Total association | 0.60(0.49,0.76) | 100 |  | 0.72(0.61,0.84) | 100 |
| Controlled direct association | 0.61(0.49,0.77) | 84.5(71.6,100.7) |  | 0.72(0.60,0.85) | 86.5(66.3,110.0) |
| Reference interaction | 0.00(-0.06,0.12) | 0.7(-42.7,15.9) |  | 0.01(-0.06,0.09) | -3.0(-39.3,19.8) |
| Mediated interaction | 0.01(-0.03,0.03) | -2.0(-7.8,12.3) |  | 0.00(-0.02,0.02) | -0.2(-5.9,9.0) |
| Pure indirect association | 0.93(0.87,0.97) | 18.2(6.9,40.0) |  | 0.95(0.92,0.99) | 16.8(6.3,32.8) |
| Proportion mediated(%) | 16.2(3.0,49.1) | |  | 16.6(4.7,38.3) | |

^a^ adjusted for age, gender, education, marital status, smoking, alcohol consumption, sleep duration, nap duration, hypertension, diabetes, dyslipidemia, chronic lung diseases, cardiovascular disease, and arthritis.

PEF, peak expiratory flow; PEFR, peak expiratory flow rate; HR, hazard ratio; CI, confidence interval.

**eTable 4 Cognition function lag two years as mediator for the association between lung function indicators and sarcopenia**

| Association component | PEF | |  | PEFR | |
| --- | --- | --- | --- | --- | --- |
|  | HR(95%CI)^a^ | Percentage of excess association (95% CI) |  | HR(95%CI^)a^ | Percentage of excess association (95% CI) |
| Total association | 0.70(0.62,0.78) | 100 |  | 0.76(0.68,0.84) | 100 |
| Controlled direct association | 0.72(0.63,0.80) | 87.9(79.1,95.9) |  | 0.77(0.69,0.86) | 87.1(74.6,97.9) |
| Reference interaction | -0.01(-0.03,0.03) | 1.8(-11.8,9.6) |  | 0.00(-0.03,0.03) | 0.1(-14.9,13.3) |
| Mediated interaction | 0.01(-0.01,0.02) | -1.8(-5.0,2.2) |  | 0.00(-0.01,0.01) | -1.1(-4.7,3.1) |
| Pure indirect association | 0.96(0.94,0.98) | 12.2(6.4,20.9) |  | 0.97(0.95,0.98) | 13.9(7.6,22.9) |
| Proportion mediated(%) | 10.3(4.3,20.1) | |  | 12.8(5.4,24.3) | |

^a^ adjusted for age, gender, education, marital status, smoking, alcohol consumption, sleep duration, nap duration, hypertension, diabetes, dyslipidemia, chronic lung diseases, cardiovascular disease, and arthritis.

PEF, peak expiratory flow; PEFR, peak expiratory flow rate; HR, hazard ratio; CI, confidence interval.

**eTable 5 Cognition function as mediator for the association between lung function indicators and sarcopenia after excluding participants with chronic lung diseases at baseline**

| Association component | PEF | |  | PEFR | |
| --- | --- | --- | --- | --- | --- |
|  | HR(95%CI)^a^ | Percentage of excess association (95% CI) |  | HR(95%CI)^a^ | Percentage of excess association (95% CI) |
| Total association | 0.71(0.63,0.81) | 100 |  | 0.77(0.69,0.86) | 100 |
| Controlled direct association | 0.73(0.64,0.83) | 87.6(75.1,97.6) |  | 0.78(0.69,0.88) | 88.3(71.6,103.1) |
| Reference interaction | 0.00(-0.03,0.04) | -0.6(-18.7,10.5) |  | 0.01(-0.03,0.06) | -4.8(-32.0,11.4) |
| Mediated interaction | 0.00(-0.01,0.02) | -1.0(-6.3,5.3) |  | 0.00(-0.01,0.01) | 0.6(-5.4,9.4) |
| Pure indirect association | 0.96(0.94,0.98) | 14.0(5.9,25.6) |  | 0.96(0.94,0.98) | 15.9(7.6,30.9) |
| Proportion mediated(%) | 13.0(4.3,27.7) | |  | 16.5(6.1,37.0) | |

a adjusted for age, gender, education, marital status, smoking, alcohol consumption, sleep duration, nap duration, hypertension, diabetes, dyslipidemia, chronic lung diseases, cardiovascular disease, and arthritis.

PEF, peak expiratory flow; PEFR, peak expiratory flow rate; HR, hazard ratio; CI, confidence interval.

**eTable 6 Cognition function as mediator for the association between lung function indicators and sarcopenia after excluding participants with PEF <60 L/min at baseline**

| Association component | PEF | |  | PEFR | |
| --- | --- | --- | --- | --- | --- |
|  | HR(95%CI)^a^ | Percentage of excess association (95% CI) |  | HR(95%CI)^a^ | Percentage of excess association (95% CI) |
| Total association | 0.68(0.59,0.77) | 100 |  | 0.73(0.65,0.82) | 100 |
| Controlled direct association | 0.70(0.61,0.80) | 85.8(74.2,93.7) |  | 0.75(0.66,0.85) | 84.1(70.3,96.4) |
| Reference interaction | -0.01(-0.04,0.03) | 3.2(-9.4,11.7) |  | -0.01(-0.04,0.04) | 1.9(-17.4,15.6) |
| Mediated interaction | 0.01(-0.04,0.03) | -3.1(-7.8,2.1) |  | 0.01(-0.01,0.02) | -2.0(-7.7,4.5) |
| Pure indirect association | 0.95(0.93,0.98) | 14.1(7.1,24.9) |  | 0.96(0.93,0.98) | 16.0(7.6,28.1) |
| Proportion mediated(%) | 11.0(3.5,22.5) | |  | 14.1(5.6,30.5) | |

^a^ adjusted for age, gender, education, marital status, smoking, alcohol consumption, sleep duration, nap duration, hypertension, diabetes, dyslipidemia, chronic lung diseases, cardiovascular disease, and arthritis.

PEF, peak expiratory flow; PEFR, peak expiratory flow rate; HR, hazard ratio; CI, confidence interval.

**eTable 7 Cognition function as mediator for the association between lung function indicators and sarcopenia after excluding events within in the first two years of follow-up**

| Association component | PEF | |  | PEFR | |
| --- | --- | --- | --- | --- | --- |
|  | HR(95%CI)^a^ | Percentage of excess association (95% CI) |  | HR(95%CI)^a^ | Percentage of excess association (95% CI) |
| Total association | 0.74(0.62,0.88) | 100 |  | 0.81(0.70,0.96) | 100 |
| Controlled direct association | 0.77(0.64,0.91) | 89.1(66.9,103.4) |  | 0.83(0.72,0.97) | 87.9(62.6,110.6) |
| Reference interaction | 0.00(-0.03,0.05) | 1.5(-27.1,13.1) |  | 0.01(-0.02,0.06) | -3.6(-60.8,18.6) |
| Mediated interaction | 0.00(-0.01,0.02) | -1.8(-9.4,8.6) |  | 0.00(-0.02,0.02) | 0.6(-11.2,24.9) |
| Pure indirect association | 0.97(0.94,0.99) | 11.3(-0.8,33.6) |  | 0.97(0.95,0.99) | 15.1(1.14,54.5) |
| Proportion mediated(%) | 9.5(-3.8,32.9) | |  | 15.7(-0.7,64.7) | |

^a^ adjusted for age, gender, education, marital status, smoking, alcohol consumption, sleep duration, nap duration, hypertension, diabetes, dyslipidemia, chronic lung diseases, cardiovascular disease, and arthritis.

PEF, peak expiratory flow; PEFR, peak expiratory flow rate; HR, hazard ratio; CI, confidence interval.
